# The Endoplasmic Reticulum Coat Protein II Transport Machinery Coordinates Cellular Lipid Secretion and Cholesterol Biosynthesis* [Fn FN2]

**DOI:** 10.1074/jbc.M113.479980

**Published:** 2013-12-13

**Authors:** Lee G. D. Fryer, Bethan Jones, Emma J. Duncan, Claire E. Hutchison, Tozen Ozkan, Paul A. Williams, Olivia Alder, Max Nieuwdorp, Anna K. Townley, Arjen R. Mensenkamp, David J. Stephens, Geesje M. Dallinga-Thie, Carol C. Shoulders

**Affiliations:** From the ‡ Endocrinology Centre, William Harvey Research Institute, Queen Mary University of London and Barts and The London School of Medicine and Dentistry, Charterhouse Square, London EC1M 6BQ, United Kingdom,; § Uro-Oncology Research Group, Cancer Research UK Cambridge Institute, Li Ka Shing Centre, Robinson Way, Cambridge CB2 ORE, United Kingdom,; the ¶ Medical Research Council Clinical Sciences Centre, Imperial College London, London W12 0NN, United Kingdom,; the ‖ Department of Vascular Medicine, Academic Medical Center, 1105 AZ Amsterdam, The Netherlands, and; the ** School of Medical Sciences, Department of Biochemistry, Bristol University, Bristol BS8 1TD, United Kingdom

**Keywords:** Apolipoproteins, Cholesterol Regulation, Endoplasmic Reticulum (ER), Lipoprotein Secretion, Transcriptomics

## Abstract

Triglycerides and cholesterol are essential for life in most organisms. Triglycerides serve as the principal energy storage depot and, where vascular systems exist, as a means of energy transport. Cholesterol is essential for the functional integrity of all cellular membrane systems. The endoplasmic reticulum is the site of secretory lipoprotein production and *de novo* cholesterol synthesis, yet little is known about how these activities are coordinated with each other or with the activity of the COPII machinery, which transports endoplasmic reticulum cargo to the Golgi. The Sar1B component of this machinery is mutated in chylomicron retention disorder, indicating that this Sar1 isoform secures delivery of dietary lipids into the circulation. However, it is not known why some patients with chylomicron retention disorder develop hepatic steatosis, despite impaired intestinal fat malabsorption, and why very severe hypocholesterolemia develops in this condition. Here, we show that Sar1B also promotes hepatic apolipoprotein (apo) B lipoprotein secretion and that this promoting activity is coordinated with the processes regulating apoB expression and the transfer of triglycerides/cholesterol moieties onto this large lipid transport protein. We also show that although Sar1A antagonizes the lipoprotein secretion-promoting activity of Sar1B, both isoforms modulate the expression of genes encoding cholesterol biosynthetic enzymes and the synthesis of cholesterol *de novo*. These results not only establish that Sar1B promotes the secretion of hepatic lipids but also adds regulation of cholesterol synthesis to Sar1B's repertoire of transport functions.

## Teaser

**Background:** Sar1 mediates the onward transport of ER cargo.

**Results:** Sar1B promotes VLDL secretion, whereas Sar1A antagonizes this activity, and a deficit of both reduces cholesterol biosynthesis.

**Conclusion:** Sar1B independently of and through its lipoprotein secretion function promotes the expression of genes regulating cholesterol biosynthesis.

**Significance:** Sar1B-mediated transport activities contribute to both the functional integrity of the ER membrane and blood cholesterol levels.

## Introduction

Whole-body triglyceride and cholesterol homeostasis are of fundamental importance to human health. Their failure manifests in multiple diseases, ranging from life-threatening conditions in infancy (1–4), to severe coronary heart disease in young adults (5, 6), and to indolent disorders of middle and old age (7–11). In higher organisms, the ability of tissues to accommodate large fluctuations in dietary triglyceride and cholesterol intake is well developed, involving cross-talk between the cellular processes that govern the delivery of these lipids into the circulation via triglyceride-rich, apoB-containing lipoproteins, their uptake, *de novo* lipogenesis, and cholesterol biosynthesis (12). From the study of familial hypobetalipoproteinemia (OMIM +107730) and abetalipoproteinemia (OMIM 200100), it is evident that both apolipoprotein (apo)B and the microsomal triglyceride transfer protein (MTTP)^4^ are obligatory for the production of chylomicrons and very low density lipoproteins (VLDL) within the endoplasmic reticulum (ER) of enterocytes and hepatocytes, respectively (1, 13–15). Moreover, human liver, in contrast to the intestine, uses apoB100 rather than the shorter apoB48 for exporting lipid into the circulation (12). Sar1B, a coat protein II (COPII) component, has also been shown to be central to the lipid economy by the discovery that its mutations cause the rare recessive disorder chylomicron retention disease (CMRD) (OMIM 246700) (2). However, it is not clear why this GTPase is obligatory for delivery of chylomicrons into the circulation and whether this relates to the usage of apoB48 rather than apoB100 for their production or insufficient Sar1A in the intestine of CMRD patients to compensate for loss of Sar1B function.

It is also not known why some CMRD children develop hepatic steatosis (16–18), despite severe intestinal fat malabsorption; and it is very odd that affected individuals develop severe hypocholesterolemia (2, 16–18), because therapeutic reduction of intestinal cholesterol absorption only modestly affects circulating cholesterol levels, due to compensatory rises in endogenous *de novo* cholesterol biosynthesis (12, 19, 20). Indeed, Western-type diets provide ∼400 mg of cholesterol per day, and our bodies synthesize ∼1 g *de novo* (21, 22). Hence, blood cholesterol levels reflect both dietary and endogenously synthesized cholesterol.

Cholesterol synthesis is a multistep reaction that is thought to occur in virtually all nucleated cells (23). In this context, it may be relevant that *SAR1B* expression has been detected in multiple tissues (2); most of the enzymes synthesizing cholesterol *de novo* reside in the ER membrane (24–28), including HMG-CoA reductase (29), the target of statins, a highly successful class of cholesterol-lowering drugs (30, 31). Hepatic biosynthesis may be especially sensitive to intestinal cholesterol absorption because of the liver's central position in directing cholesterol into VLDL or bile (32). After uptake by enterocytes, cholesterol is packed with triglycerides into chylomicrons and secreted into the lymph. In the circulation, the triglycerides are rapidly hydrolyzed, and the released glycerol and fatty acids taken up by peripheral tissues, whereas the cholesterol-enriched, highly atherogenic remnant particles are captured by the liver (33, 34). From internal cellular endosomal compartments, the cholesterol may be returned to the plasma membrane (35) or be transported to the ER (36, 37).

In ER membranes, cholesterol may suppress the activities of one or more cholesterol biosynthetic enzymes or activate acyl-CoA:cholesterol acyltransferase (ACAT) 2 (38–41). It may also impede binding between the COPII protein Sec24 and the sterol regulatory element-binding protein (SREBP) escort protein Scap (42, 43), thereby blocking export of the ER membrane-bound Scap-SREBP2 complex to the Golgi apparatus (44). Here, SREBP2 undergoes proteolytic cleavage, culminating in the release of its amino-terminal domain (45), known to transcriptionally activate genes regulating cholesterol homeostasis (46–50). However, it is not known whether the Sar1B component of the COPII transport machinery contributes to the ER export of SREBP2, *de novo* cholesterol synthesis, or the secretion of cholesteryl esters via VLDL.

Studies with Sar1 bound to nonhydrolysable analogues of GTP and a GTP-restricted form of Sar1 (*i.e.* Sar1:H79G) have defined several aspects of the COPII vesicle assembly process (51–54). In outline, Sar1-GDP is recruited to the cytosolic face of the ER membrane. Here, the guanine-nucleotide exchange protein Sec12 swaps the GDP on Sar1 for GTP. Next, the Sar1-GTP recruits Sec23/24 to the ER membrane to form a pre-budding complex that captures both ER cargo and Sec13/31 (55, 56), culminating in polymerization of the COPII coat and fission of the COPII vesicle from the ER, along with its captured cargo. This process, including the sorting of ER cargo into COPII pre-budding complexes, is coupled to the hydrolysis of Sar1's GTP; Sec12 continually reactivates Sar1, promoting continued pre-budding complex assembly and cargo capture, whereas unchecked GTP hydrolysis triggers coat disassembly (52–55).

In CMRD, most *SAR1B* mutations alter an amino acid in Sar1B's GDP/GTP binding pocket (2, 17, 18), suggesting that the intestinal fat malabsorption defect is attributable to breakdown/absence of the Sar1 GTPase cycle that normally secures capture of nascent chylomicrons into COPII pre-budding complexes and their subsequent onward transport. However, it is not known whether this breakdown relates to a specific aspect(s) of the apoB lipoprotein/COPII vesicle working relationship, and it is unclear to what extent Sar1B's homologue Sar1A can compensate for loss of Sar1B in this partnership. Additionally, there are no data regarding the contributions of the Sar1A- and Sar1B-GTPase cycles in securing VLDL secretion and cholesterol homeostasis.

Here, we demonstrate that ubiquitously expressed Sar1B (2) acts at two control points crucial to cholesterol metabolism. First, Sar1B promotes hepatic apoB100-lipoprotein secretion (as well as apoB48-lipoprotein secretion), and this is paired with expression changes in *APOB* and *MTTP*, but not of genes involved in triglyceride synthesis. The apoB lipoprotein secreting activity of Sar1B is antagonized by Sar1A. This is more pronounced for the more triglyceride-rich apoB lipoproteins, indicating that only Sar1B has the attributes to mediate the assembly of COPII pre-budding complexes of the right structures and composition to secure export of such nascent apoB lipoproteins and hence efficient delivery of triglyceride and cholesterol moieties into the circulation. Second, deficiency of Sar1B, but not Sar1A, in apoB lipoprotein-producing cells reduces ER export of Srebp2, whereas aggregate levels of Sar1 determine the rate of *de novo* cholesterol synthesis. The study indicates that Sar1B, both independently of and through its promotion of apoB-mediated lipid secretion activities, contributes to the regulation of cellular, as well as plasma, triglyceride and cholesterol levels.

## EXPERIMENTAL PROCEDURES

### 

#### 

##### Production of Stable Cell Lines, Knockdown, and Tissue Culture

Human *SAR1A* and *SAR1B* sequences were PCR-amplified from Marathon-ready human liver cDNA (Clontech) using gene-specific primers. FLAG epitope tags (Asp-Tyr-Lys-Asp_4_-Lys) were fused in-frame to the carboxyl termini of cDNAs and juxtaposed to a terminator codon. PCR and appropriate restriction sites were used to manipulate the *SAR1* sequences. Site-directed mutagenesis was performed by a standard two-step PCR-based strategy (57). All constructs were sequenced before use. Transfection of pcDNA4/TO vectors into McArdle-RH7777 (58) and CHO (Invitrogen) cell lines stably transformed with pcDNA6/TR was performed with either FuGENE (Roche Applied Science) or Lipofectamine Plus Reagent (Invitrogen). Individual clones were picked, expanded, and screened for recombinant protein expression using serial dilutions of cell lysates, anti-FLAG M2 antibody (Sigma-Aldrich), a goat anti-mouse IgG horseradish peroxidase-conjugated secondary antibody (Bio-Rad), and Femto-enhanced chemiluminescence (Pierce). Blots were scanned on a flatbed scanner and immunoreactive products quantified using Adobe Photoshop. Cell lines (two per construct) were selected to have comparable levels of recombinant protein expression.

Knockdowns were performed with pre-designed siRNA (Qiagen) using HiPerFect transfection reagent (Qiagen) according to the manufacturer's fast forward protocol. Volumes of transfection reagents and concentrations of siRNA were optimized for each pre-designed FlexiTube siRNA (Qiagen). The best results were obtained using a final concentration of 5 nm siRNA. For the double-knockdown experiments, 5 nm of each isoform was used. The scrambled siRNA sequence was 5′-UUCUCCGAACGUGUCACGUdTdT-3′ (Qiagen).

McArdle-RH7777 cells were cultured at 37 °C, 5% CO_2_ in DMEM containing 20% heat-inactivated FCS (Labtech International), 4500 mg/liter d-glucose, 2 mm l-glutamine (Invitrogen), and 1% penicillin/streptomycin (Invitrogen). CHO cell lines were cultured in Ham's F-12 media (Sigma) containing 10% heat-inactivated FCS, 2 mm l-glutamine, and 1% penicillin/streptomycin. Blasticidin (10 μg/ml; Sigma) and Zeocin (250 μg/ml; Invitrogen) were added to the media of cells stably transfected with the tetracycline repressor (pcDNA6/TR) and operon (pcDNA4/TO) vectors (Invitrogen).

##### Metabolic Labeling, Immunoprecipitations, and Sucrose Gradient Ultracentrifugation of ApoB Lipoproteins

Prior to labeling for 1 h with 125 Ci/ml l-[^35^S]methionine (ICN Flow) at 37 °C, cells were incubated with complete DMEM containing 1 μg/ml tetracycline between 24 and 48 h and then in serum-free media supplemented with 0.8 mm oleic acid (Sigma) complexed to 3% fatty acid-free BSA overnight and cysteine/methionine-free media (Invitrogen) containing oleate for 30 min. After removal of label, incubations were continued in serum-free media supplemented with 0.8 mm oleic acid complexed to 3% fatty acid-free BSA for 2 h. Conditioned media were cleared by low speed centrifugation and concentrated to 500 μl in Amicon Ultra-4 centrifugal filter units with Ultracel-30 membranes (Millipore). ApoB was immunoprecipitated by standard methodology (59, 60), using saturating concentrations of goat polyclonal antibody against human apoB (AB742; Chemicon International), as described (13, 59, 60). The radioactivity in apoB48 and apo100 was quantified by phosphorimager analysis. Three samples were collected per stable cell line per experiment.

For total protein secretion measurements, cells were incubated in methionine-free medium for 45 min at 37 °C and labeled for 1 h with 125 Ci/ml l-[^35^S]methionine (ICN Flow). After removal of label, incubations were continued in serum-free medium for 2 h. Cell media were harvested, mixed with protease inhibitors (Calbiochem), and cleared by low speed centrifugation. Proteins were precipitated by incubation with TCA for 15 min on ice. Precipitates were washed in ice-cold acetone and resuspended in I.P. buffer containing protease inhibitors (Calbiochem).

Densities of secreted ^35^S-labeled apoB lipoproteins were determined by gradient ultracentrifugation. The sucrose gradients were formed as described (13), except that the gradients were ultracentrifuged at 35,400 rpm in a Beckman SW41 rotor and unloaded in 12 fractions. ApoB was recovered from each fraction by immunoprecipitation and size-fractionated by SDS-PAGE, and the amount of radioactivity in apoB48 and full-length apoB100 was quantified by phosphorimager analysis. The density profile of the gradient was determined using an Abbe refractometer.

##### Immunoblot Analysis of Endogenous Protein Expression

Cell lysates were generated using RIPA buffer (Sigma) and a mixture of protease inhibitors (Roche Applied Science). Proteins were size-fractionated on either 10 or 4–12% gradient NuPage BisTris precast gels (Invitrogen) and transferred to nitrocellulose membranes (Whatman). Membranes were blocked in 5% (w/v) nonfat milk powder before probing with the specified antibodies. The antibodies used were as follows: rabbit polyclonal anti-Hmgcs1 antibody (Proteintech); anti-Lss antibody (Proteintech); anti-Dhcr7 antibody (Abcam); anti-ACSL5 (Proteintech); anti-Sec13 antibody (Abcam); anti-Sec31a antibody, anti-Sar1a antibody, anti-Sar1b antibody, and anti-Srebf2 antibody (Proteintech); anti-ATF6 (Abcam); and mouse monoclonal β-actin or β-tubulin antibodies (Sigma and Abcam). Immunoreactive products were visualized and quantified after labeling with appropriate fluorescent dye-labeled secondary antibodies (LI-COR Biosciences) using the Odyssey imaging system (LI-COR). Apparent molecular masses were estimated using the Novex Sharp Protein Standard (Invitrogen) and the band sizing application in Odyssey software (LI-COR Biosciences).

##### RT-qPCR Analyses in Cell Lines

Pre-designed gene primers were purchased from Qiagen (Quantitect primer assay), unless stated otherwise (supplemental Table S1). Total RNA was isolated from cell lysates using the RNeasyPlus kit (Qiagen). Cells were homogenized using QIAshredder columns (Qiagen). RNA samples were quantified by a NanoDrop 1000 spectrophotometer (Thermo Scientific). RT-qPCRs were performed in triplicate (with a no RT control) with SYBR Green kits from Qiagen or Stratagene, as recommended by the manufacturers. Data were analyzed with either SDS 2.3 software (Applied Biosystems) or MxPro (Stratagene). Gene expression levels, normalized to *Ppia*, were calculated by the relative 2^−ΔΔ^*^Ct^* method (61).

##### RNA Analyses of Human Small Intestine Biopsies and Liver Samples

After an overnight fast, gastroduodenoscopy was performed, and small intestinal (jejunal) biopsies were taken near the Treitz' ligament in 17 obese male subjects with no history of disease or any medication use (*i.e.* treatment-naive). To minimize variability in sample processing and handling, biopsy samples were collected immediately and snap-frozen in liquid nitrogen. All subsequent sample processing was performed by the same individual using the same standardized protocol. Tissue was homogenized in MagNA Pure LC RNA Isolation Tissue Lysis Buffer (Roche Applied Science), using a MagNALyser instrument (Roche Applied Science), and using parameters that were optimized on comparable samples prior to commencing the study. Total RNA was prepared using TRIzol according to the manufacturer's manual (Invitrogen), quantified by a NanoDrop 1000 spectrophotometer (Thermo Scientific), and reverse-transcribed into cDNA using a cDNA synthesis kit from Bio-Rad. RT-qPCR was performed in triplicate with 50 ng of cDNA, 200 nm of each primer, and 7.5 μl of SYBR Green mix (CE Biotech). PCRs were performed on a CFX system (Bio-Rad). Gene expression levels normalized to 36B4 were calculated by the relative 2^−ΔΔ^*^Ct^* method (61). The study was approved by the Research Ethics Committee at Amsterdam Medical Center, Amsterdam, The Netherlands, and all participants gave written informed consent.

RT-qPCR on human liver RNA (First Choice Human Total RNA, Applied Biosytems) was performed with a SYBR Green kit (Qiagen). Correlations between mRNA levels in human liver samples (62, 63) were computed using R2, a Genomics Analysis Visualization Platform. *p* values were corrected for multiple testing using the formula implemented in R2: *t* = *R*/sqrt(1 − *R*^2)/(*n* − 2), where *R* is the correlation coefficient, and *n* denotes the number of samples.

##### Microarray Gene Analyses

A minimum of three technical replicates were used for each of two Sar1B:H79G-FLAG cell lines, two control cell lines, two Sar1B-FLAG cell lines, and one Sar1A-FLAG cell line. Cells were cultured in complete DMEM containing blasticidin (10 μg/ml; Sigma) and Zeocin (250 μg/ml; Invitrogen). Twenty four hours prior to harvesting, 1 μg/ml tetracycline was added to the culture media. Total RNA was extracted with the RNeasy Plus mini kit (Qiagen). RNA quality was verified (*e.g.* flat baseline between 18 S and 28 S ribosomal RNA peaks; RIN score >7) using the RNA 6000 Ladder (Ambion), RNA 6000 NanoChip (Agilent), plus an Agilent 2100 bioanalyzer (Agilent Technologies, Germany). cDNA was synthesized from total RNA using the one-cycle eukaryotic target labeling assay kit (Invitrogen and Affymetrix). Double-stranded cDNA was purified with the GeneChip® sample cleanup module. Biotin-labeled cRNA was prepared using the BioArray High Yield RNA transcript labeling kit (Enzo) and purified with the *in vi*t*ro* transcription cRNA GeneChip® sample cleanup module. RNA quality was verified with the Agilent Bioanalyzer 2100 system and by hybridization to test arrays (Affymetrix). Biotinylated cRNA (20 μg) was hybridized to GeneChip rat genome 230 2.0 arrays (Affymetrix) containing 31,099 probe sets. All cRNA samples were prepared using the same standard protocol.

Data analyses were performed through the CARMAweb (Comprehensive R-based Microarray Analysis web service). Raw intensity values were normalized using the Loess normalization, Affymetrix MAS 5 and summarization algorithms. Differences in probe-set values were determined on the normalized data using the significance analysis of microarray (SAM) algorithm (64) at specified false discovery rates (FDR). These were determined using the “Benjamini and Hochberg” correction, with cutoff values for significance determined by a tuning parameter, the “Δ value.” The number of Δ values was set at 50. Data are available at NCBI Gene Expression Omnibus (GEO accession number GSE52969). The differentially expressed probe sets were examined for enrichment of functionally related Gene Ontology terms using the Database for Annotation, Visualization, and Integrated Discovery (DAVID; david.abcc.ncifcrf.gov) (65, 66).

##### Lipid Synthesis and Secretion

Cells were seeded at a density of 1 × 10^4^ cells/cm^2^ in 9-cm plates and grown for 2 days in DMEM containing 20% FCS, 1% penicillin/streptomycin, and 1 mm glutamine at 37 °C under 5% CO_2_. Lipid synthesis was measured over the final 5 h of the incubation by the addition of 2 mm [^14^C]acetate (sodium salt; Sigma) (0.8 μCi/ml). Cell media were aspirated for analysis of lipid secretion. Cells were washed in PBS and harvested by trypsinization and centrifugation. Cell pellets were washed in PBS and lipids extracted by addition of 4 ml of chloroform/methanol (2:1). Water (1 ml) was added to the extract and mixed by vortexing. The suspension was centrifuged for 5 min, and the lower solvent layer was taken for analysis. The upper aqueous layer was re-extracted with a further 4 ml of chloroform/methanol, and the two extractions were combined. This was dried down under N_2_ and resuspended in a minimal volume of hexane (30 μl). Cell medium (4 ml) was extracted as above, using 16 ml of chloroform/methanol (2:1). Lipids were separated by thin layer chromatography on Silica Gel 60 plates (VWR) using petroleum ether/diethyl ether/acetic acid (60:40:1) as the mobile phase and visualized by iodine staining. Each run included standards of cholesterol, fatty acids, triglycerides and cholesterol esters. Lipid synthesis was measured by scraping of spots into vials followed by liquid scintillation counting. Plates were also visualized by autoradiography to determine that no major lipid product was missed.

##### Statistics

All data are expressed as means ± S.E. Statistical analyses were performed using unpaired Student's *t* test or one-way analysis of variance followed by the Bonferroni post hoc test using PASW Statistics version 18.0 software (SPSS, IBM), where appropriate. Figures were generated using Graphpad Prism 4.

## RESULTS

### 

#### 

##### Characterization of the Effects of Sar1 Isoforms on ApoB Lipoprotein Secretion

To establish whether Sar1B promotes only the secretion of apoB48-containing lipoproteins, we stably transfected McArdle-RH7777 cells that can secrete both apoB48 and apoB100 lipoproteins with constructs encoding Sar1A-FLAG, Sar1B-FLAG, or constitutively active Sar1-FLAG (*i.e.* Sar1A:H79G and Sar1B:H79G) isoforms that cannot hydrolyze GTP to support completion of COPII vesicle assembly (67). Despite several attempts, we failed to stably transfect McArdle-RH7777 cells with Sar1A:H79G, potentially because *Sar1a* mRNA is the predominant species expressed in these cells (11.6-fold higher than *Sar1b*). The Sar1A cell lines contained 5.26 ± 0.48- and 2.41 ± 0.48-fold, respectively, more recombinant Sar1 than the Sar1B and Sar1B:H79G cell lines.

**FIGURE 1.**
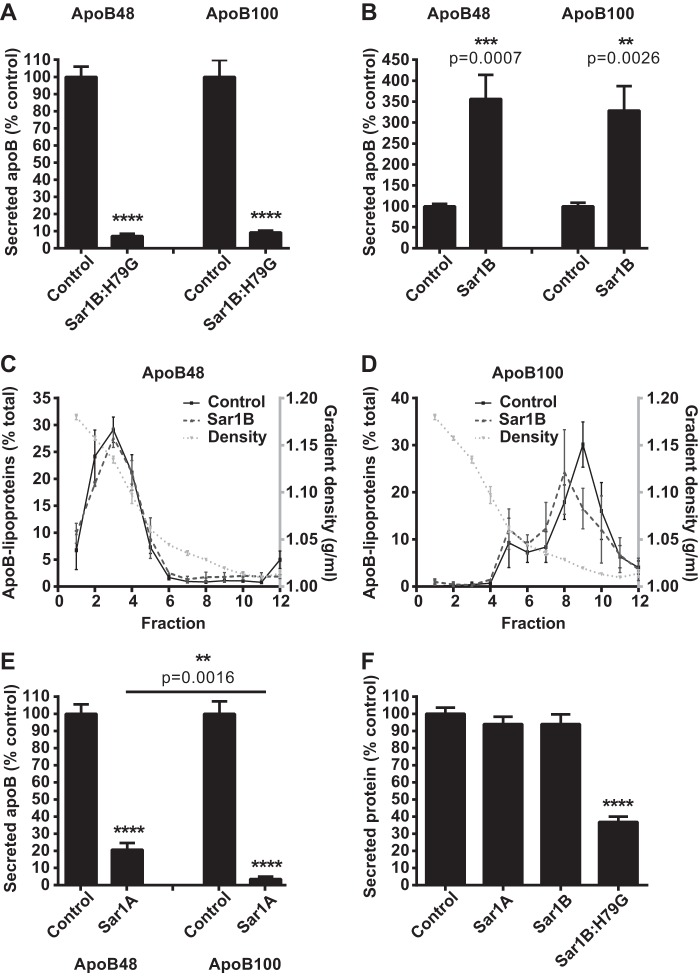
**Sar1A and Sar1B have opposite effects on apoB48- and apoB100-containing lipoprotein secretion.** *A* and *B*, McArdle-RH7777 cells were labeled with l-[^35^S]methionine for 60 min and chased for 120 min. The l-^35^S-labeled apoB in the cell media was immunoprecipitated, analyzed by SDS-PAGE, and visualized by phosphorimaging. Data (mean ± S.E.) are from four independent experiments.****, *p* < 0.0001 *versus* cells with empty vector control. ***, *p* < 0.001 *versus* cells with empty vector control. **, *p* < 0.01 *versus* cells with empty vector control. *C* and *D*, densities of fractions containing secreted l-^35^S-labeled apoB48-containing (*C*) and l-^35^S-labeled apoB100-containing (*D*) lipoproteins were determined gravimetrically. Data (mean ± S.E.) are from three independent experiments. *E*, secreted l-^35^S-labeled apoB from two independent Sar1A-FLAG cell lines, quantified as in *A* and *B*. ****, *p* < 0.0001 *versus* cells with empty vector control. **, *p* < 0.01 *versus* apoB100 lipoproteins. *F*, total l-[^35^S]methionine in secreted protein fraction. Data (mean ± S.E.) are from five independent experiments. ****, *p* < 0.0001 *versus* cells with empty vector control.

Sar1B:H79G overexpression virtually abolished the secretion of both apoB48 and apoB100 lipoproteins (Fig. 1*A*). By contrast, Sar1B overexpression increased their secretion by >3-fold (Fig. 1*B*). Their densities were not decreased (Fig. 1, *C* and *D*) with both the apoB48- and apoB100-containing lipoproteins exhibiting their expected densities (13). Sar1A overexpression markedly reduced apoB lipoprotein secretion (Fig. 1*E*); however, the effect was more pronounced for apoB100 than apoB48 (secretion 3.49 ± 1.5% (apoB100) and 20.65 ± 3.97% (apoB48) of control cells, respectively; *p* = 0.0016 for difference). Total protein secretion was also decreased in the Sar1B:H79G cell lines (Fig. 1*F*), but this reduction was significantly less than that observed for apoB48/100 (Fig. 1*A*). Collectively, these data show that Sar1B promotes secretion of both apoB48 and apoB100 lipoproteins, whereas Sar1A overexpression has the opposite effect, preferentially blocking the secretion of the more lipid-laden particles.

**FIGURE 2.**
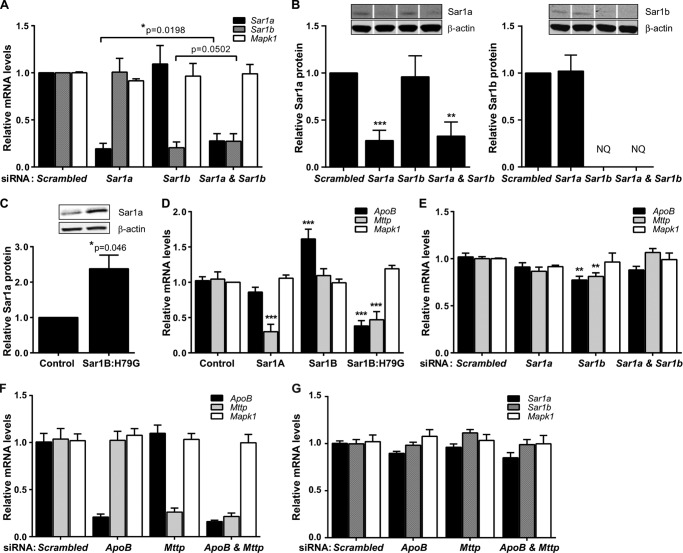
**Effect of constitutively active Sar1B and native Sar1 expression on *ApoB* and *Mttp* expression in McArdle-RH7777 cells.** *A* and *B*, isoform-specific effects of *Sar1a* and *Sar1b* knockdown on transcript (*A*) and protein (*B*) analyzed by RT-qPCR and quantitative Western blot analysis. *NQ*, not quantified due to undetectable levels of Sar1b in some cell lysates. *White line* separators indicate that noncontiguous lanes from the same gel are shown. *A*, *, *p* < 0.05 *versus Sar1a* + *Sar1b* knockdown. *B*, ***, *p* < 0.001 *versus* scrambled control siRNA; **, *p* < 0.01 *versus* scrambled control siRNA. *C*, Sar1a protein in stably overexpressing Sar1B:H79G and empty vector control cells. *, *p* < 0.05 *versus* cells with empty vector control. *D*, Sar1 isoform-specific effects on *ApoB* and *Mttp* mRNA levels in overexpressing cell lines, analyzed by RT-qPCR. ***, *p* < 0.001 *versus* cells with empty vector control. *E, Sar1b* knockdown specifically reduces *ApoB* and *Mttp* mRNA, analyzed by RT-qPCR. **, *p* < 0.01 *versus* scrambled control siRNA. *F* and *G, ApoB* and *Mttp* knockdown (*F*) does not decrease *Sar1a* and *Sar1b* mRNA (*G*), analyzed by RT-qPCR. *A–G*, data (mean ± S.E.) are from at least three independent experiments.

To determine whether Sar1 also had isoform-specific effects on the expression of *ApoB* and *Mttp*, we measured the levels of their RNAs by RT-qPCR in our McArdle-RH7777 cell lines plus McArdle-RH7777 cells transfected with *Sar1* siRNAs. Individual knockdown of the *Sar1* isoforms were isoform-specific, and there were no increases in the mRNA (Fig. 2*A*) or protein (Fig. 2*B*) levels of the nontargeted isoform. The amounts of Sar1a protein in cells transfected with *Sar1a* and *Sar1a* plus *Sar1b* siRNAs were 28.16 ± 6.36 and 32.88 ± 8.70%, respectively, of that in cells transfected with the scrambled siRNA control (Fig. 2*B*). *Sar1b* knockdown also substantially reduced in Sar1b protein and in the majority of samples to undetectable levels (Fig. 2*B*). This was due to the Western blot analysis returning weaker signals for Sar1b than Sar1a, consistent with RT-qPCR data (*Sar1b* mRNA 11.6-fold lower than *Sar1a* mRNA), plus the efficiency of *Sar1b* knockdown in reducing Sar1b protein (≥70% in the rare knockdown sample where it could be quantified).

Although constitutively active Sar1B increased *Sar1a* mRNA (4.22 ± 0.29-fold compared with control cells) and protein (Fig. 2*C*), it reduced *ApoB* and *Mttp* mRNA (Fig. 2*D*). Levels of the control gene, *Mapk1*, were not altered (Fig. 2*D*). In the complementary analyses, native Sar1B overexpression increased *ApoB* mRNA (Fig. 2*D*), while having no effect on *Mttp* (Fig. 2*D*) and *Sar1a* (1.27 ± 0.03-fold, nonsignificant) mRNAs; and *Sar1b* knockdown reduced *ApoB* and *Mttp* mRNA (Fig. 2*E*) and had no significant effect on *Sar1a* mRNA (Fig. 2*A*). In the control analysis, *ApoB* and *Mttp* knockdown (Fig. 2*F*) did not reduce *Sar1b* mRNA (Fig. 2*G*). Interestingly, we found that Sar1A overexpression decreased *Mttp* mRNA levels, although *Sar1a* knockdown did not increase *Mttp* mRNA (Fig. 2*E*). However, in the double knockdown experiment, decreased Sar1a did prevent the fall in *Mttp* (and *apoB*) associated with *Sar1b* knockdown (Fig. 2*E*). Thus, collectively these data indicate that Sar1 has isoform-specific effects on *ApoB* and *Mttp* mRNA levels. Moreover, because a higher level of MTTP activity is required for producing apoB100 than apoB48 lipoproteins (15), the observed changes in *ApoB* and *Mttp* expression may form part of the adaptive responses to Sar1-induced changes in apoB lipoprotein secretion.

Because clinical studies have indicated that *SAR1B* mutations affect chylomicron secretion and, despite intestinal fat malabsorption, some children with CMRD develop fatty liver (2, 16–18), we examined relative *SAR1A* and *SAR1B* mRNA levels in human intestinal biopsies and liver samples. In addition, we examined the correlation between *SAR1*, *APOB*, and *MTTP* mRNA in publically available human liver gene expression datasets.

We found that *SAR1B* mRNA levels were 239 ± 84- and 3-fold, respectively, higher than *SAR1A* in intestinal mucosal biopsies (17 donors) and a pooled (three donors) liver sample. By contrast, the predominant *SEC23* (A) and *SEC31* (A) isoforms expressed in these two tissues displayed more comparable mean fold differences; in the intestinal biopsies, *SEC23A* and *SEC31A* were 5.0 ± 0.9- and 43 ± 12-fold higher than their respective *B* isoforms; and in the pooled liver sample, the corresponding values were 3.2 and 29.4.

We also found positive correlations between hepatic *SAR1B*, but not *SAR1A*, mRNA levels and *APOB*/*MTTP* mRNA. Thus, in a large dataset comprising 427 individual liver samples, the correlation coefficient (*R*) between *SAR1B* and *APOB* mRNA was 0.301 (*p* = 8.3 × 10^−9^), and in the second dataset, comprising 75 samples, this result was replicated (*R* = 0.292, *p* = 0.01). Likewise, *R* values between *SAR1B* and *MTTP* mRNA were 0.268 (*p* = 2.5 × 10^−8^) in dataset 1 and 0.346 (*p* = 2.4 × 10^−3^) in dataset 2. *SAR1A* displayed no positive correlation with either *APOB* or *MTTP* mRNA levels; *R* values for the *SAR1A*/*APOB* correlations were 0.003 (dataset 1, *p* = NS) and −0.012 (dataset 2, *p* = NS) and between *SAR1A* and *MTTP*, −0.179 (*p* = 2.3 × 10^−4^) and −0.019 (*p* = NS). Notably, the weak inverse correlation between *SAR1A* and *MTTP* mRNA levels in dataset 1 is consistent with the effect of Sar1A overexpression on *Mttp* mRNA levels in McArdle-RH7777 cells (Fig. 2*D*), plus the *Mttp* mRNA findings in the set of *Sar1* knockdown experiments (Fig. 2*E*).

Thus, collectively our results (*e.g.* Fig. 2, *D* and *E*) and the previous characterization of the COPII vesicle assembly process (51, 54, 56, 68) are consistent with a model whereby the expression of *APOB* and *MTTP*, which are obligatory for apoB lipoprotein production in the ER lumen (12, 69, 70), are modulated by the expression of Sar1B, which facilitates the export of nascent apoB lipoproteins (and potentially other associated cargo) out of the ER in COPII-coated vesicles.

##### Characterizing Sar1 Effects on Gene Expression and de Novo Cholesterol Biosynthesis

In view of the lipid transport functions of apoB, we determined whether constitutively active Sar1B altered the expression of genes regulating triglyceride, fatty acid, and cholesterol homeostasis. To this end, we performed a microarray RNA analysis on the McArdle-RH7777 cell lines and validated the robustness of the results by measuring *Sec* mRNA levels via RT-qPCR in an independent experiment (supplemental Table S2). In addition, we confirmed that the respective 2.73 ± 0.15- and 2.90 ± 0.47-fold increases in *Sec13* and *Sec31a* mRNA associated with Sar1B:H79G overexpression (supplemental Table S2) were congruent with estimated rises in Sec13 (1.7 ± 0.20-fold) and Sec31a (4.72 ± 0.94-fold) protein.

**FIGURE 3.**
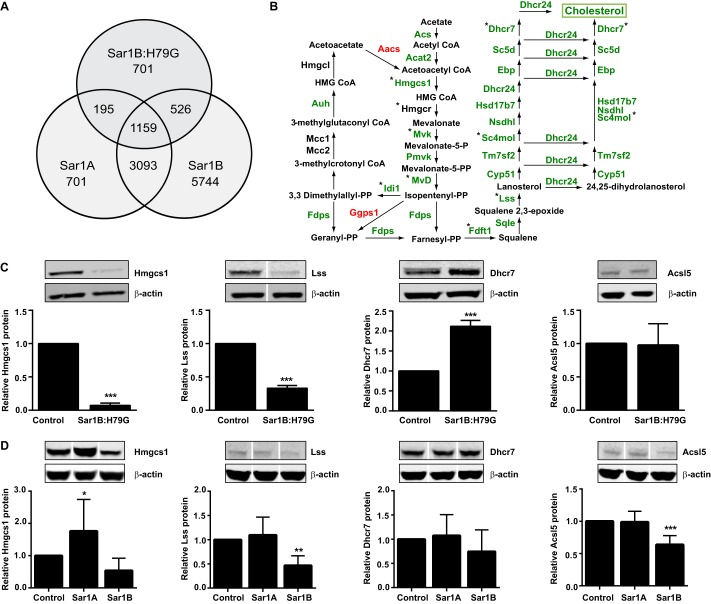
**Sar1B:H79G decreases cholesterol biosynthesis gene expression in McArdle-RH7777 cells, independently of reduced *Apob* and *Mttp* expression.** *A*, expression values of 701 probe sets were significantly altered (FDR, 0.000215; Δ 6.8) by Sar1B:H79G but not Sar1A (FDR 0.009, Δ1.9) or Sar1B (FDR 0.015, Δ 2.0) overexpression. *B, green* labeling depicts reduced mRNA levels in stably overexpressing Sar1B:H79G cells. *, expression values are verified by RT-qPCR. Note: in stably overexpressing Sar1A and Sar1B cells, mRNA levels of these genes were not reduced (supplemental Table S5). *C* and *D*, relative protein levels of three representative enzymes on the cholesterol biosynthetic pathway (Hmgcs1, Lss, Dhcr7) and a control enzyme, Acsl5, in stably transfected cells overexpressing Sar1B:H79G (*C*), Sar1A, and Sar1B (*D*). *C* and *D* data (mean ± S.E.) are from at least three independent experiments. ***, *p* < 0.001 *versus* cells with empty vector control; **, *p* < 0.01 *versus* cells with empty vector control. *White line* separators indicate that noncontiguous lanes from the same gel are shown.

We identified 701 probe sets that had altered expression values (FDR <0.000215) in the Sar1B:H79G cell lines only (Fig. 3*A*). In the control analyses (*i.e.* native Sar1 overexpression), fewer probe sets were uniquely differentially expressed as follows: three in the Sar1A- and 70 in the Sar1B-overexpressing cell lines (supplemental Fig. S1). The Sar1B:H79G probe set contained an over-representation of genes assigned the Gene Ontology terms “Cholesterol and Lipid Biosynthetic and Metabolic Processes” (Table 1), although neither the Sar1A nor Sar1B datasets did (supplemental Tables S3 and S4). We therefore collated the expression values for genes active in fatty acid, triglyceride, and cholesterol metabolism, and verified the mRNA levels of representative genes in an independent experiment by RT-qPCR (supplemental Table S5).

**TABLE 1:** **Enrichment of gene ontology biological processes terms in the Sar1B:H79G uniquely differentially expressed gene-probe list** The uniquely differentially expressed Sar1B:H79G dataset (see also Fig. 3*A*) was analysed for enrichment of functionally related Gene Ontology (GO) terms using DAVID (65, 66).

Cluster description	GO terms in cluster	Differentially expressed genes (*n*)	Enrichment score	*p* value range
Regulation of molecular function and catalytic activity	0065009; 0050790; 0051336	32	3.70	2.1 × 10^−5^-1.5 × 10^−3^
Cholesterol and lipid biosynthetic and metabolic processes	0006695; 0008203; 0016126; 0016125; 0044255; 0006629; 0008610; 0006694; 0008202	32	2.16	7.3 × 10^−4^-8.6 × 10^−3^
Amine catabolic processes	0009310; 0044270; 0009065; 0006527; 0009063; 0009064	9	1.59	3.3 × 10^−3^-2.8 × 10^−2^
Regulation of GTPase activity	0043087	7	1.54	6.7 × 10^−3^
Response to nutrient levels and extracellular stimulus	0031667; 0009991	10	1.49	8.1 × 10^−3^-1.0 × 10^−2^
Regulation of hydrolase activity	0051336; 0043087; 0032313; 0032483; 0032482; 0032318	13	1.38	1.5 × 10^−3^-4.5 × 10^−2^
Response to glucocorticoid and corticosteroid stimulus	0051384; 0031960	5	1.3	3.0 × 10^−2^-4.4 × 10^−2^
Amine catabolic processes	0009310; 0044270; 0042133; 0006575; 0042219; 0042135	16	1.28	3.3 × 10^−3^-3.4 × 10^−2^
Negative regulation of catalytic activity	0043086; 0033673; 0006469; 0051348	9	1.21	3.4 × 10^−3^-3.0 × 10^−2^
Neurogenesis	0022008; 0048699; 0030182; 0048812; 0048667; 0007409	21	1.18	1.0 × 10^−2^-4.0 × 10^−2^
Response to stress	0006950; 0009605	51	1.04	1.5 × 10^−2^-1.7 × 10^−2^
Response to endogenous stimulus	0009719	21	0.90	1.2 × 10^−2^
Protein import into nucleus	0006606; 0051170	7	0.87	2.5 × 10^−2^-2.8 × 10^−2^
Alcohol metabolic process	0006066	23	0.76	3.1 × 10^−4^

Despite reduced secretion of apoB lipoproteins from the Sar1B:H79G and Sar1A cell lines (Fig. 1, *A* and *E*), and increased apoB-secretion from Sar1B cells (Fig. 1*B*), we found that the levels of most transcripts encoding proteins active in fatty acid and triglyceride metabolism were unaltered by increases in Sar1A expression in the Sar1A and Sar1B:H79G (Fig. 2*C*) cell lines and by Sar1B overexpression (supplemental Table S5). By contrast, constitutively active Sar1B decreased the levels of most mRNAs encoding enzymes on the cholesterol biosynthetic pathway (Fig. 3*B* and supplemental Table S5), whereas overexpression of native Sar1A and -B did not (supplemental Table S5).

Western blot analysis of Sar1B:H79G cell lysates revealed markedly reduced levels of Hmgcs1 and Lss protein (Fig. 3*C*, *1st* and *2nd panels*), whereas Dhcr7, which converts 7-dehydrocholesterol to cholesterol and 7-dehydrodesmostrel to 24-dehydrocholesterol (Fig. 3*B*), was increased >2-fold (Fig. 3*C*, *3rd panel*). In a control analysis, Sar1B:H79G did not alter RNA (supplemental Table S5) or protein levels of Acsl5 (Fig. 3*C*, *4th panel*), a key regulator of fatty acid metabolism (71). Furthermore, Sar1A overexpression, which also markedly impaired apoB lipoprotein secretion (Fig. 1*E*), did not reduce Hmgcs1 and Lss protein (Fig. 3*D*) or increase Dhcr7 protein (Fig. 3*D*). Rather Hmgcs1 protein was increased (Fig. 3*D*, *1st panel*).

Sar1B overexpression had a variable impact on Hmgcs1 protein levels and only significantly reduced Lss (Fig. 3*D*). However, no increase in Dhcr7 protein was observed, and in marked contrast to Sar1A and Sar1B:H79G overexpression, Ascl5 protein was reduced (Fig. 3*D*). Thus, these data indicate that overexpression of Sar1A, Sar1B, and Sar1B:H79G have distinct effects on Hmgcs1, Dhcr7, and Acsl5 protein levels and that only Sar1B:H79G significantly reduced levels of mRNA encoding cholesterol-biosynthetic enzymes (supplemental Table S5).

In a further control experiment, we found that knockdown of *ApoB* and *Mttp* did not significantly reduce mRNA levels of cholesterol pathway-encoding enzymes (supplemental Fig. S2). Thus, these data substantiate the evidence from the *Sar1A* gene expression (supplemental Table S5) and apoB secretion results (Fig. 1*E*) that partial ablation of cholesterol/cholesteryl ester secretion via the apoB-containing lipoprotein axis (40, 69, 72, 73) was not sufficient by itself to explain the Sar1B:H79G-mediated reductions in the levels of transcripts encoding cholesterol biosynthetic enzymes (Fig. 3*B* and supplemental Table S5).

**FIGURE 4.**
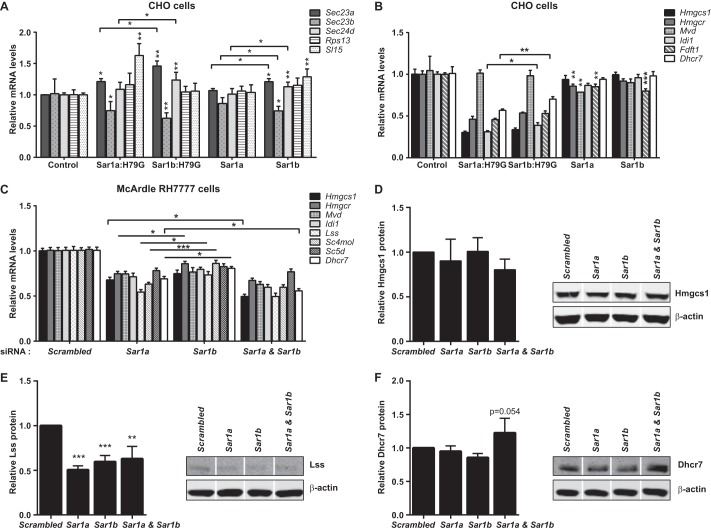
**No Sar1 isoform-specific effects on cholesterol biosynthesis gene expression in non-apoB and apoB lipoprotein-secreting cells.** *A* and *B*, relative mRNA levels of COPII and control genes (*A*) and representative cholesterol genes (*B*) in CHO cells stably overexpressing constitutively active Sar1A or Sar1B and native Sar1A or Sar1B, analyzed by RT-qPCR. *A*, *, *p* < 0.05 *versus* cells with empty vector control or cells expressing Sar1b:H79G or Sar1b. **, *p* < 0.01 *versus* cells with empty vector control. *B*, *, *p* < 0.05 *versus* cells expressing Sar1b:H79G; **, *p* < 0.01 *versus* cells with empty vector control or cells expressing Sar1b:H79G; ***, *p* < 0.001 *versus* cells with empty vector control. *C, Sar1a* and *Sar1b* knockdown in McArdle-RH7777 cells reduce levels of representative mRNA encoding cholesterol biosynthesis enzymes. *, *p* < 0.05 *versus Sar1b* (with or without *Sar1a*) siRNA; ***, *p* < 0.001 *versus Sar1b* siRNA. *D–F*, relative protein levels of Hmgcs1 (*D*), Lss (*E*), and Dhcr7 (*F*) in McArdle-RH7777 cells transfected with *Sar1a* and *Sar1b* siRNAs. *White line* separators indicate that noncontiguous lanes from the same gel are shown. *E*, ***, *p* < 0.001 *versus* scrambled control siRNA; **, *p* < 0.01 *versus* scrambled control siRNA. *A–F*, data (mean ± S.E.) are from three independent experiments.

CHO cells do not have the machinery to produce apoB-containing lipoproteins (74). We therefore used them to explore further the impact of native and constitutively active Sar1 overexpression on the levels of mRNAs encoding cholesterol biosynthetic enzymes. *Sar1A* mRNA levels were respectively increased by 18.68 ± 2.04- and 22.13 ± 1.12-fold in the Sar1A and Sar1A:H79G cell lines; and the corresponding *Sar1B* values for the Sar1B and Sar1B:H79G cell lines were 21.17 ± 2.87 and 38.08 ± 3.71. *Sec* and control gene expression was much more modestly affected (Fig. 4*A*). In these non-apoB lipoprotein-producing cell lines, the overexpression of native Sar1A and Sar1B reduced cholesterol biosynthesis gene expression, but to a lesser extent than the control genes (Fig. 4, *A* and *B*). By contrast, constitutively active Sar1A or Sar1B overexpression markedly reduced mRNA levels of the cholesterol gene set (Fig. 4*B*). Hence, these data support the proposition that the reduced levels of most transcripts encoding enzymes for cholesterol biosynthesis in the McArdle-RH7777 cell lines expressing Sar1B:H79G are partially separable from the apoB lipoprotein secretion defect.

The gene expression data from CHO cell line experiments (Fig. 4*B*) also suggested that both Sar1 isoforms modulate the levels of mRNAs encoding cholesterol biosynthetic enzymes. We examined this further in McArdle-RH7777 cells by determining the effects of *Sar1a* and *Sar1b* knockdown on mRNA levels of eight representative members of this gene set. All were decreased (Fig. 4*C*). *Sar1b* knockdown generally produced smaller reductions than knockdown of *Sar1a*, the predominant species in McArdle-RH7777 cells. In the double knockdown, *Hmgcs1* and *Dhcr7* mRNA levels were significantly lower than in the individual knockdowns (Fig. 4*C*). However, Hmgcs1 protein levels were not significantly decreased by either individual or combined knockdown of *Sar1a* and *Sar1b* (Fig. 4*D*). In comparison, Lss protein was significantly reduced by both individual and combined *Sar1a* and S*ar1b* knockdown (Fig. 4*E*). Combined (not individual) *Sar1a* and *Sar1b* knockdown also produced a nonsignificant rise in Dhcr7 (Fig. 4*F*, *p* = 0.054 for difference from scrambled siRNA control). Thus, collectively, these data indicate that both Sar1A and Sar1B modulate the levels of mRNAs encoding cholesterol biosynthetic enzymes, supporting the CHO cell findings (Fig. 4*B*). Substantiating this conclusion, we also found that in human liver samples, *SAR1A* and *SAR1B* mRNA levels exhibited no isoform-specific correlations with 20 of the 21 mRNA species encoding cholesterol biosynthetic enzymes (supplemental Table S6). The only isoform-specific correlation was between that of *SAR1B* and *SC5DL* (sterol-C5-desaturase), and both of these genes had expression values that exhibited positive correlation with *APOB* and *MTTP* mRNA levels (supplemental Table S6).

**FIGURE 5.**
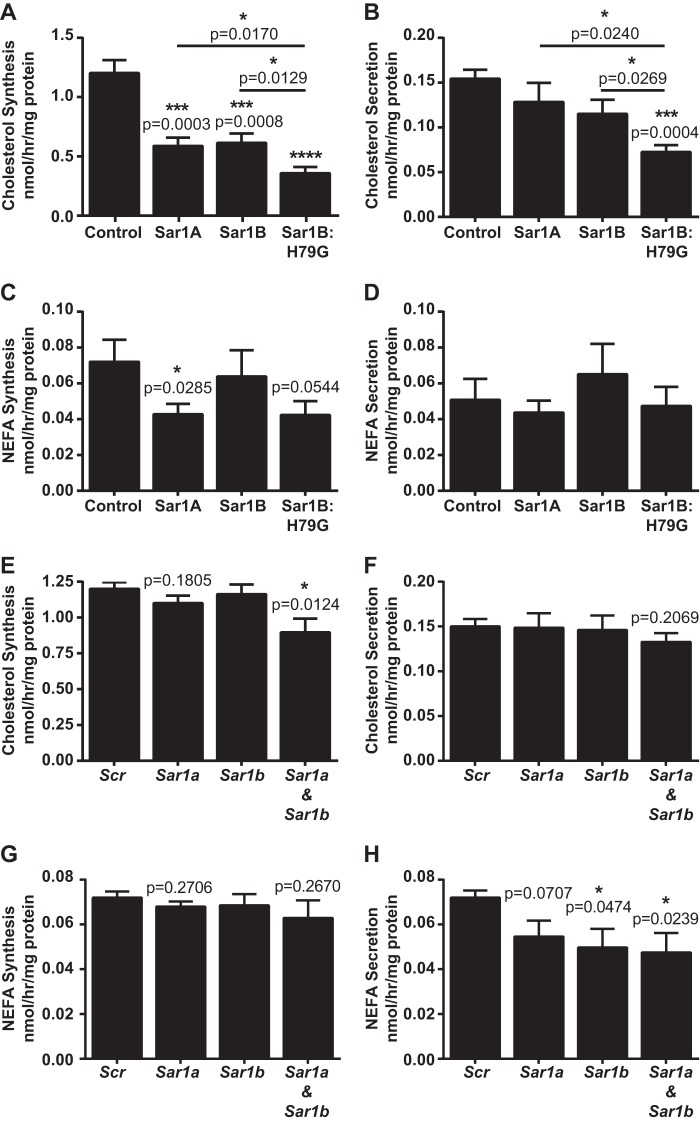
**Sar1B:H79G and combined Sar1a and Sar1b deficiency reduce *de novo* cholesterol synthesis to greater extents than cholesterol secretion.** *A–H*, McArdle-RH7777 cells stably overexpressing specified recombinant Sar1 protein or control McArdle-RH7777 cells transfected with specified siRNA were labeled with 2 mm [^14^C]acetate for 5 h. *A* and *B*, [^14^C]cholesterol in the cell (*A*) and media (*B*) of specified cell lines. *C* and *D*, [^14^C]NEFA contents in the cell (*C*) and media (*D*) of specified cell lines. *E* and *F*, [^14^C]cholesterol contents in the cell (*E*) and media (*F*) of McArdle-RH7777 cells transfected with specified siRNAs. *G* and *H*, [^14^C]NEFA contents in the cell (*G*) and media (*H*) of cells transfected with specified siRNAs. Data (mean ± S.E.) are from three (*A–D*) or four (*E–H*) independent experiments, *Scr*, scrambled siRNA. *A* and *B*, ***, *p* < 0.001 *versus* cells with empty vector control; ****, *p* < 0.0001 *versus* cells with empty vector control; *, *p* < 0.05 *versus* cells expressing Sar1B:H79G. *C*, *, *p* < 0.05 *versus* cells with empty vector control. *E* and *H*, *, *p* < 0.5 *versus* scrambled control siRNA.

Next, we wanted to establish whether the reductions in “cholesterol” gene expression (Figs. 3*B* and 4*C*) and associated protein changes (Figs. 3*C* and 4, *D–F*) translated into reduced *de novo* cholesterol synthesis. As estimated from the incorporation of [^14^C]acetate into cholesterol, Sar1B:H79G overexpression markedly reduced the rate of *de novo* cholesterol synthesis (Fig. 5*A*). Additionally, [^14^C]cholesterol secretion was reduced (Fig. 5*B*) but to a lesser extent than *de novo* cholesterol synthesis. The estimated rate of incorporation of [^14^C]acetate into nonesterified fatty acids (NEFA) was also reduced (Fig. 5*C*), but this was to a much lesser degree than *de novo* cholesterol synthesis, and there was no corresponding reduction in ^14^C-labeled NEFA secretion (Fig. 5*D*). Consistent with comparable transcription profiles (supplemental Table S5), the Sar1A and Sar1B cell lines displayed similar rates of *de novo* cholesterol synthesis (Fig. 5*A*), both of which were significantly higher than that of the Sar1B:H79G cell lines.

Individual knockdown of *Sar1a* and *Sar1b* did not reduce the estimated rates of *de novo* cholesterol synthesis (Fig. 5*E*). By contrast, combined *Sar1a* and *Sar1b* knockdown had a significant impact reducing cellular [^14^C]cholesterol by 25.33 ± 8.04% (Fig. 5*E*), and this was paired with a small, albeit nonsignificant, reduction in the estimated rate of [^14^C]cholesterol secretion (Fig. 5*F*). The estimated rate of incorporation of [^14^C]acetate into NEFA was also reduced by combined *Sar1a* and *Sar1b* knockdown but by no more than individual *Sar1a* and *Sar1b* knockdowns (Fig. 5, *G* and *H*). Thus, these data, combined with the results in Figs. 3, *B–D*, and 4, *B–F*, and previous findings (52–55), indicate that an insufficiency of Sar1 to support pre-budding complex assembly or the sequestration of certain cargo into pre-budding COPII complexes containing constitutively active Sar1 reduce cholesterol biosynthesis.

Because cholesterol synthesis may both affect and be affected by the processing of Srebp2 in the Golgi apparatus, and COPII vesicles transport the ∼123-kDa ER membrane-bound precursor Srepb2 polypeptide to the Golgi (44, 45), we examined the effects of Sar1B:H79G overexpression and *Sar1a* and *Sar1b* knockdown, on Srepb2 expression. Additionally, we determined cellular levels of ATF6, a non-sterol-dependent transcription factor. The ATF6 precursor resides in the ER membrane (75) and is activated by the same proteases as Srebp2 in the Golgi (76, 77) to specifically regulate ER stress-inducible genes (78).

**FIGURE 6.**
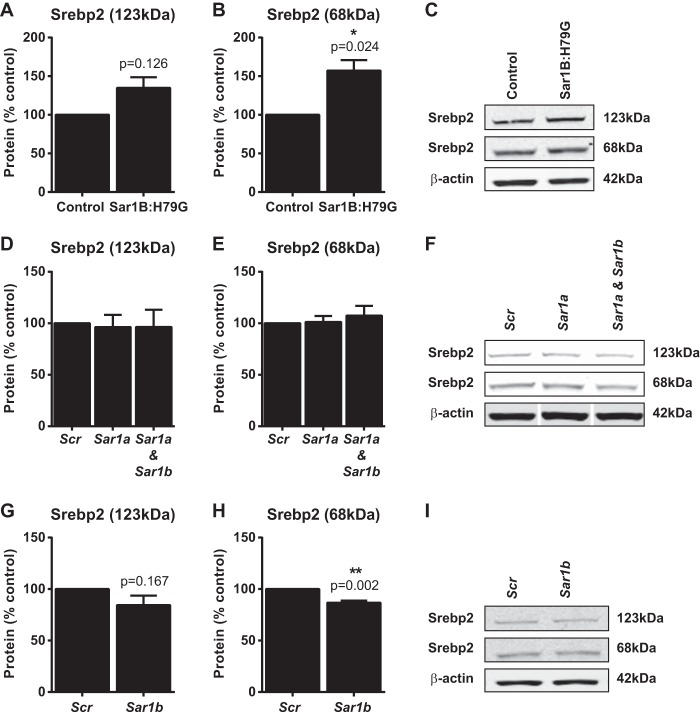
**Constitutively active Sar1B increases Srebp2 levels, whereas Sar1b deficiency decreases Srepb2 processing in McArdle-RH7777 cells.** *A–C*, Srebp2 protein in stably overexpressing Sar1B:H79G and empty vector control cells. *D–F, Sar1a* and *Sar1a*+ *Sar1b* knockdown do not alter the levels of the 123-kDa precursor (*D* and *F*) or of the 68-kDa processed (*E* and *F*) Srebp2 protein. *G–I, Sar1b* knockdown decreases (*H* and *I*) the level of 68-kDa processed Srebp2 protein. *A–I*, data (mean ± S.E.) are from a minimum of three independent experiments. *B*, *, *p* < 0.05 *versus* cells with empty vector control. *H*, **, *p* < 0.01 *versus* scrambled control siRNA. In Western blot analyses, *white line* separators indicate that noncontiguous lanes from the same gel are shown. *D–I, Scr*, scrambled siRNA.

We found that Sar1B:H79G overexpression led to a small, but nonsignificant, rise in the level of precursor Srebp2 (Fig. 6*A*) and a significant increase in the level of the 68-kDa processed polypeptide (Fig. 6, *B* and *C*). Levels of the control protein ATF6 were not altered (supplemental Fig. S3). Thus, these results indicate that there was sufficient endogenous Sar1 in the Sar1B:H79G cell lines (Fig. 2*C*) to support the ER export of both Srebp2 and ATF6 precursors.

Sar1a knockdown did not reduce either Srebp2 protein levels (Fig. 6, *D–F*) or *Scap* and *Srebp2* mRNA expression (supplemental Table S7). Likewise, combined *Sar1a* and *Sar1b* knockdown did not decrease Srebp2 protein levels or its processing (Fig. 6, *D–F*). By contrast, knockdown of *Sar1b* (minority *Sar1* species in McArdle-RH7777 cells), which had no impact on *de novo* cholesterol synthesis (Fig. 5*E*), significantly reduced (by 13.2 ± 1.93% *versus* scrambled, *p* = 0.002 for difference) the level of processed Srebp2 (Fig. 6, *G–I*) and *Scap* mRNA (supplemental Table S7). In the control analysis, *Sar1b* knockdown did not reduce the amount of the non-sterol-responsive transcription factor ATF6 (supplemental Fig. S3).

## DISCUSSION

This study was designed to examine the interplay between apoB-containing lipoprotein secretion and *de novo* cholesterol synthesis and their reliance on the common and individual activities of the two Sar1 isoforms. We provide strong evidence that Sar1B promotes the secretion of triglyceride-rich, apoB-containing lipoproteins from the liver, which would neatly explain the counter-intuitive observation that some CMRD children develop hepatic steatosis, despite severe intestinal fat malabsorption (16–18). Moreover, we show that Sar1A antagonizes Sar1B's lipoprotein secretion-promoting activity. We also establish that *SAR1B* is the predominantly expressed isoform in human jejunum and liver, two organs that substantially contribute to circulating levels of cholesterol (12, 79, 80), and that Sar1 deficiency, as well as constitutively active Sar1, decrease the levels of transcripts encoding cholesterol biosynthetic enzymes and *de novo* cholesterol synthesis. The results, which help explain the unique nature of the very severe hypocholesterolemia phenotype (*i.e.* reduced levels of all cholesterol-carrying lipoproteins) that features in CMRD (16, 18, 81), indicate that Sar1B makes a substantial contribution to the regulatory processes controlling ER and blood cholesterol levels.

As touched upon in the Introduction, there is a close spatial and temporal association between apoB and the ER membrane, extending from its point of entry to its co-translational assembly into a nascent lipoprotein (82). This transformation is promoted by triglyceride and cholesterol synthesis (72, 82–88), plus the conversion of membrane-bound cholesterol to cholesteryl esters (40, 69, 73, 83, 89), whereas the transport of newly assembled apoB lipoproteins from their sites of synthesis in the ER to the Golgi apparatus is mediated by the COPII transport machinery.

With the results from this study, we can now conclude that only Sar1B, and not Sar1A, can secure the efficient secretion of nascent, triglyceride-rich, apoB-containing lipoproteins and that it does so whether they are assembled around apoB48 or apoB100 and in the liver or intestine. Indeed, we found that Sar1A antagonizes Sar1B's apoB lipoprotein secretion-promoting activity, and this antagonism was more pronounced for larger and more lipid-laden apoB lipoproteins. Thus, these data suggest that Sar1B may have a unique capacity to promote the production of COPII vesicles of the size required to secure the ER export of triglyceride-rich, apoB-containing lipoproteins. This proposition fits well with existing knowledge. First, a few abnormally small chylomicrons (diameter 63 ± 19 nm) have been found within the abluminal space of CMRD patients' enterocytes (90), implying that chylomicrons (which are assembled around apoB48) exit the ER in standard sized COPII vesicles so long as they remain small. Second, in both of the two unrelated CMRD patients investigated, VLDL (which is assembled around apoB100) was markedly reduced (91). Third, in rat hepatocytes, VLDL formed around either apoB48 or apoB100 buds from the ER in larger sized COPII vesicles than do protein cargo (92). Moreover, a significant proportion of the apoB100 lipoproteins are found in larger sized COPII vesicles than apoB48 particles (92), likely reflecting that apoB100 has more neutral lipid-binding motifs than apoB48. Indeed, assembling apoB100 lipoproteins not only require more lipid than apoB48 to attain a soluble conformation, they will, with all other things being equal (*e.g.* lipid availability), bind considerably more lipid than apoB48 (12, 93, 94).

Although early investigations on CMRD patient samples revealed that both intestinal and hepatic apoB-containing lipoproteins were produced in this condition (81, 90), a re-appraisal of these data is long overdue given the new information on Sar1 (53–55) and the results reported herein. Thus, the totality of the data indicates that the removal of apoB lipoproteins from their sites of synthesis in the ER lumen may facilitate the apoB lipoprotein assembly process itself. We note, for example, that Sar1B's lipoprotein secretion-promoting activity was associated with a marked increase in *ApoB* expression (Fig. 2*D*) but not of genes regulating triglyceride synthesis (supplemental Table S5) and that Sar1B promoted the production of increased numbers of apoB lipoproteins rather than of more lipid-laden ones (Fig. 1, *C* and *D*). In comparison, Sar1B mutations in CMRD culminate in the production of abnormally large chylomicrons (90) and a lipid phenotype resembling that observed in the recessively inherited lipoprotein production disorder, abetalipoproteinemia; specifically, enterocytes from CMRD patients contain large cytosolic lipid droplets, even in the fasting state (90). Thus, loss of Sar1B's lipoprotein secretion-promoting activity appears to impair apoB lipoprotein production, albeit to a lesser extent than the impairment that occurs in abetalipoproteinemia.

Tantalizing clues as to how Sar1B could promote the assembly of specialist pre-budding complexes for mediating the onward transport of nascent apoB lipoproteins have emerged from two *in vitro* studies (56, 95). The first showed that Sar1B relaxes its host membrane to a greater extent that Sar1A, implying the former may promote assembly of large radius pre-budding complexes more readily than the latter, all other factors being equal (95). The second, incidentally found during study of the pathogenic Sec23A-F382L mutation (56), is that Sar1B-Sec23-Sec24 pre-budding complexes have lower affinity for Sec13/Sec31A than equivalent Sar1A-Sec23-Sec24 complexes, reducing Sec23-mediated hydrolysis of GTP on Sar1. The implication, which seems entirely consistent with their study's morphological analyses, is that Sar1B serves to delay, relative to Sar1A, COPII coat disassembly, thereby tilting the balance toward continued COPII vesicle expansion. In fact, a prolonged COPII vesicle assembly process could permit elongating apoB polypeptides to commandeer its ER-to-Golgi transport vesicle early on in the lipoprotein production process (∼10–15 min for apoB100 (13)), well before it undergoes the huge increase in size that occurs during transformation of hepatic and intestinal apoB into lipoproteins.

We found that relative to *SAR1A*, *SAR1B* mRNA levels are much higher in the upper gastrointestinal mucosa than in liver. Nonetheless, it is apparent that *SAR1B* contributes in a major way to hepatic lipid metabolism given the combination of our apoB lipoprotein studies (*e.g.* Figs. 1*B* and 2, *D* and *E*), the finding of hepatic steatosis in some children with CMRD (16, 17, 91), the highly significant positive correlations between *SAR1B*, *APOB*, and *MTTP* mRNA in human liver samples, and the finding that partial *Sar1b* knockdown reduced Srebp2 processing (Fig. 6*H*) paired with the expected corresponding reduction in levels of mRNAs encoding enzymes active in cholesterol biosynthesis (Fig. 4*C*).

We found that Sar1 had no isoform-specific effects on the expression of genes regulating triglyceride or fatty acid metabolism (supplemental Table S5). Moreover, even though constitutively active Sar1B blocked apoB lipoprotein secretion (Fig. 1*A*), it did not reduce the expression of most members of these two gene sets, suggesting that the intracellular transport of Srebp1, the best characterized transcription factor regulating fatty acid and triglyceride gene expression (47, 96–99), was maintained in our Sar1B:H79G cell lines. Indeed, because our stable cell lines overexpressing Sar1B:H79G induced a compensatory rise of endogenous Sar1A (Fig. 2*C*), we very likely created the means for allowing the onward transport of some ER cargo, while facilitating detection of Sar1B-specific phenotypes.

Although we found that native Sar1A and Sar1B overexpression had no, or minimal, effect on the levels of mRNA encoding cholesterol biosynthetic enzymes (supplemental Table S5 and Fig. 4*B*), they did reduce *de novo* cholesterol biosynthesis, albeit to a much lesser extent than Sar1B:H79G overexpression. It seems particularly pertinent that Sar1A overexpression, which markedly decreased apoB lipoprotein secretion (Fig. 1*E*), was associated with increased Hmgcs1 protein levels, and Sar1B overexpression was associated with reduced Lss protein. Conceivably, this counter-intuitive rise in Hmgcs1 protein (but not mRNA) could reflect a response to the diversion of acetyl-CoA (Fig. 3*B*) away from the cholesterol biosynthesis pathway toward an alternative metabolic pathway(s), for example, pathways promoting the acetylation of ER resident and ER-transiting proteins (100) and/or histone tails (101). In comparison, the reduction in Lss protein associated with Sar1B overexpression, however it is mediated, may reflect part of an adaptive response that serves to reduce flux through the cholesterol biosynthetic pathway.

The observation that Sar1B:H79G overexpression reduced the levels of most transcripts encoding cholesterol biosynthetic enzymes (Fig. 3*B* and supplemental Table S5) and that this reduction was paired with increased, rather than decreased, Dhcr7 protein levels is of interest (Fig. 3*C*). Dhcr7 deficiency in embryonic mouse brains leads to a compensatory rise in Hmgcs1 protein (102). This reciprocal relationship also develops on Sar1B:H79G overexpression, despite it having comparable effects on *Hmgcs1* and *Dhcr7* mRNA levels (supplemental Table S5). Likewise, the expected reciprocal relationship between reduced *de novo* cholesterol synthesis and increased Srepb2 processing (Fig. 6, *B* and *C*) was sustained. However, this increased processing was insufficient to secure the expected corresponding increases in cholesterol biosynthetic gene expression, as judged by mRNA measurements.

There are several possible explanations that could account for the effect of the Sar1B:H79G mutation on the levels of mRNA encoding cholesterol metabolism genes. For instance, it could reflect reduced Srebp2-mediated activation of these genes, for example, as a result of dimerization of Srebp2 with Srebp1c and/or another transcriptional co-factor (103, 104). Next, the processes regulating stabilization of one or more of these transcripts may be compromised (105). Alternatively, it could reflect their enhanced degradation, for example, via an miRNA-mediated (106) or IRE1α-mediated (107) mechanism(s). What is clear is that the incorporation of constitutively active Sar1B into pre-budding complexes would sequester certain ER cargo as a result of delaying the completion of COPII vesicle assembly and thus the fission of COPII vesicles, along with their selected cargo, from the ER membrane (51, 52, 55). Furthermore, reducing cholesterol secretion via the apoB lipoprotein pathway does not adequately explain the reduced mRNA levels associated with expression of constitutively active Sar1 (*e.g.* supplemental Fig. S2).

We also found that reducing the level of Sar1a protein decreased the levels of mRNA encoding cholesterol biosynthetic enzymes (Fig. 4*C*), but despite this, estimated rates of *de novo* cholesterol synthesis and secretion were not reduced. Consistent with this, Srebp2 processing was not increased. Thus, our results indicate that in our cell system there was sufficient Sar1 (*i.e.* residual Sar1a protein and Sar1b) to initiate the assembly of pre-budding COPII complexes for ER export of Srebp2 and that factors other than reduced Srebp2 processing underlie the reduced mRNA levels of the cholesterol gene set. By contrast, the reduced processing of Srebp2 associated with knockdown of *Sar1b* (minority species) may explain some of the reduction in the levels of mRNA encoding cholesterol biosynthetic enzymes. Hence, the observed cholesterol/Srebp2/mRNA differences between the Sar1B:H79G overexpressing cells and cells deficient for Sar1a or Sar1b indicate that the processes regulating *de novo* cholesterol synthesis in apoB lipoprotein-producing cells may be somewhat more complex than in other cell types. Furthermore, modulation of Sar1A and Sar1B levels in such cells affects processes regulating the accessibility of *de novo* synthesized cholesterol within the ER membrane (108, 109), its subsequent transfer to and utilization in the plasma membrane (110, 111), in addition to its use in the production of apoB- and apoA1-containing lipoproteins (38–40, 112).

The observation that *Sar1* knockdown reduced *de novo* cholesterol synthesis by ∼25% is likely to be clinically relevant, especially given that this phenotype developed in cells that retained ∼30% of their total Sar1 (*i.e.* a and b) protein. It is also very striking that the severe hypocholesterolemia of CMRD differs from abetalipoproteinemia (1, 113) and familial hypobetalipoproteinemia (14) in that it is characterized by very low levels of high density lipoprotein (HDL) cholesterol, and indeed, in some cases, absence of this so-called good cholesterol-carrying lipoprotein particle (16, 18, 81, 114). HDL is formed through the efflux of plasma membrane cholesterol onto lipid-free apoAI, and cellular cholesterol levels are a major determinant of this efflux from the plasma membrane (114, 115). Therefore, it must be acknowledged that *SAR1B* mutations in CMRD very likely contribute to the low HDL cholesterol phenotype through an overall reduction in whole-body *de novo* cholesterol synthesis and that this reduction may, to a large extent, stem from those cells that have relatively low Sar1A levels or a high requirement for *de novo* synthesized cholesterol, or a combination of both.

With respect to understanding the role of hepatic *de novo* cholesterol synthesis in regulating apoB-containing lipoprotein secretion, *in vivo* data are rather sparse. One study found that liver-specific knockdown of *HMGCR* in 3- and 4-week-old mice significantly reduced plasma apoB, plus total and LDL cholesterol levels (116). Conversely, another showed that hepatic overexpression of Fdft1, which produces the first specific intermediate in the cholesterol biosynthetic pathway (Fig. 3*B*), increased *de novo* cholesterol synthesis and the production of cholesterol-enriched VLDL, culminating in markedly raised plasma levels of total and LDL cholesterol (87). Because the post-squalene cholesterol reactions (Fig. 3*B*) occur on the ER membrane (24–28), it is plausible that reduced *de novo* cholesterol synthesis may decrease ACAT activity. Indeed, Parini *et al.* (38) have shown that culturing cells in cholesterol-enriched media increases the enzymatic activity of ACAT2, the major cholesterol-esterifying enzyme in hepatocytes (39), and Temel *et al.* (40) demonstrated that ACAT2 stimulates cholesteryl ester secretion in apoB lipoproteins. Furthermore, it has been demonstrated that MTTP increases ACAT's activity by transferring its reaction products (*i.e.* cholesteryl esters) onto assembling apoB lipoproteins, thereby removing product inhibition (69). Thus, in light of these considerations, and the data presented here, it seems to make sense that in CMRD, VLDL, and LDL in the patients contain, as percentage of total cholesterol, significantly less cholesteryl ester than control samples (81), whereas their VLDL carries a larger than usual triglyceride load (16, 81, 90). More fundamentally, the cholesterol/cholesteryl ester and apoB/MTTP pathways work with each other, as well as the COPII and Srepb2/Scap machineries, to maintain the cholesterol content of the ER membrane at a concentration(s) compatible with this cellular compartment's biosynthetic organizing functions (117).

## Footnotes

4 The abbreviations used are:

MTTP: microsomal triglyceride transfer protein
ER: endoplasmic reticulum
apo: apolipoprotein
COPII: coat protein II
CMRD: chylomicron retention disease
ACAT: acyl-CoA:cholesterol acyltransferase
SREBP: sterol regulatory element-binding protein
FDR: false detection rate
NEFA: nonesterified fatty acid
BisTris: 2-[bis(2-hydroxyethyl)amino]-2-(hydroxymethyl)propane-1,3-diol
qPCR: quantitative PCR.
